# Efficient Control of Zika Virus Infection Induced by a Non-Replicating Adenovector Encoding Zika Virus NS1/NS2 Antigens Fused to the MHC Class II-Associated Invariant Chain

**DOI:** 10.3390/v13112215

**Published:** 2021-11-03

**Authors:** Loulieta Nazerai, Søren Buus, Anette Stryhn, Allan Randrup Thomsen, Jan Pravsgaard Christensen

**Affiliations:** Department of Immunology and Microbiology, University of Copenhagen, 2200 Copenhagen, Denmark; julietanaze@gmail.com (L.N.); sbuus@sund.ku.dk (S.B.); astryhn@sund.ku.dk (A.S.); athomsen@sund.ku.dk (A.R.T.)

**Keywords:** Zika virus, vaccine, NS1/NS2, CD8 T cell response, MHC-II invariant chain

## Abstract

It is generally believed that a successful Zika virus (ZIKV) vaccine should induce neutralizing antibodies against the ZIKV envelope (E) protein to efficiently halt viral infection. However, E-specific neutralizing antibodies have been implicated in a phenomenon called antibody-dependent enhancement, which represents an ongoing concern in the flavivirus-vaccinology field. In this report, we investigated the vaccination potential of replication-deficient adenoviral vectors encoding the ZIKV non-structural proteins 1 and 2 (NS1/NS2) and employed the strategy of linking the antigens to the MHC-II associated invariant chain (li) to improve immunogenicity and by inference, the level of protection. We demonstrated that li-linkage enhanced the production of anti-NS1 antibodies and induced an accelerated and prolonged polyfunctional CD8 T cell response in mice, which ultimately resulted in a high degree of protection against ZIKV infection of the CNS.

## 1. Introduction

The severe clinical consequences of Zika virus (ZIKV) infection including microcephaly in newborns and Guillain–Barre syndrome in adults, highlight the pressing need for a protective vaccine. ZIKV is a sexually transmitted and mosquito-borne pathogen belonging to the Flavivirus family along with Dengue, Yellow fever, and West Nile virus [1]. The viral genome contains one open reading frame encoding a single polypeptide, which is subsequently cleaved into three structural proteins, (capsid (C), premembrane/membrane (prM/M), and envelope (E)), and seven non-structural proteins, (NS1, NS2A, NS2B, NS3 NS4A, NS4B, and NS5) [2].

ZIKV is generally quite genetically homogenous, and although an African and Asian lineage is distinguished, all variants seem to belong to a single serotype [3], suggesting that one vaccine should suffice for general protection. There are currently no licensed human vaccines, but a handful has reached initial clinical evaluation including purified inactivated ZIKV as well as DNA, mRNA, and vector-based vaccines carrying the prM/E proteins [4,5]. Studies performed in animal models suggest that neutralizing antibodies (nAbs) targeting the E protein on the viral surface play a primary role in protection [5,6]. The E protein has three domains (DI, DII, DIII) and while all three can induce nAbs, the nAbs directed against EDIII are more potent than those against EDI and EDII. However, the presence of Abs at sub-neutralizing levels may contribute to a more severe course of subsequent infections via the phenomenon of antibody-dependent enhancement (ADE). During ADE, sub-neutralizing antibodies may form complexes with viral particles, which may subsequently be phagocytosed by cells expressing the Fcγ receptor, thus promoting viral replication even in cells that are not normally permissive to that viral infection [7]. Infection with the closely related dengue flavivirus, DENV, is a prominent example of ADE. The E proteins of DENV share 54–59% amino acid similarity with the E proteins of ZIKV [8]. The potential of pre-existing DENV immunity to cause ADE following a subsequent ZIKV infection, and vice-versa, has been demonstrated in mouse studies [9,10]. More importantly, while studies using non-human primate (NHPs) models have failed to provide unequivocal results [11,12], human clinical studies indicate that prior ZIKV infection increases the risk of severe dengue disease [13,14]. It should be noted that ADE in the context of sexual and transplacental ZIKV transmission has not yet been addressed neither in NHP models nor in human studies.

Consequently, there is a risk of failure/complications for vaccination strategies relying exclusively or primarily on the induction of anti-E ZIKV antibodies. With this in mind, alternative vaccine targets have been explored with the NS1 ZIKV protein, showing promising potential. The flavivirus NS1 is an interesting glycoprotein that can be found in both the cytosol and on the cell surface of infected cells as well as in a secreted form [15,16]. The functions of the three forms of NS1 are not entirely clear, but antibodies against the cell surface form seem to direct complement-mediated lysis of infected cells, while the intracellular form is implicated in the early stages of RNA replication [16]. The levels of the secreted NS1 early in the infection are high and proteins have been shown to accumulate not only in the supernatant, but also on the surface of uninfected cells [17,18]. The ZIKV NS1 protein may represent a target for both antibodies, and T cells and studies in mouse models, using constructs targeting this molecule, have demonstrated protective immunity [19,20]. More specifically, delivering the ZIKV NS1 with the Modified Vaccinia Ankara (MVA) vector has successfully protected immunocompetent mice from lethal ZIKV infection [19], while delivering ZIKV NS1 with the Vesicular Stomatitis Virus (VSV) vector was able to confer partial protection [21]. Moreover, combining NS1 with prME improved the protective efficiency of VSV- and Adenovirus-based vaccines in mouse models [20,21], and in a recent paper, a DNA vaccine encoding a secreted ZIKV NS1 was found to improve virus control through T-cell mediated immunity [22]. Interestingly, the immune response induced by an NS1 encoding DNA vaccine was found to be augmented by genetically fusing NS1 to the immune enhancer HSV-gD [23]. Thus, it is evident that choosing the right delivery platform and perhaps ensuring the most efficient presentation of the antigenic molecule are key for a successful vaccine against ZIKV without the risk of ADE.

In this study, we used the well-studied replication-deficient adenoviral vector platform to deliver the ZIKV non-structural proteins NS1/NS2 and employed the strategy of linking the antigens to the MHC-II associated invariant chain (li) to enhance immunogenicity [24,25,26,27,28,29]. The concept of tethering vaccine antigen to li has recently been evaluated in a clinical study and extending pre-clinical observations, it was also demonstrated to improve immunogenicity in humans [30]. Here, we show that: (I) vaccination with Ad-liNS1/NS2 is able to confer increased levels of protection against subsequent intracranial (i.c.) ZIKV challenge in immunocompetent adult mice; (II) li-linkage enhances the production of anti-NS1 antibodies and induces an accelerated ZIKV-specific CD8 T cell response that is sustained in vaccinated mice at high levels for an extended period of time; and (III) polyfunctional CD8 T cells are necessary for protection in vaccinated mice.

## 2. Materials and Methods

### 2.1. Mice

Female C57BL/6 (B6) wild type (WT) mice were obtained from Taconic farms (Ry, Denmark) at the age of 6–8 weeks old. IFN-γ–deficient mice on a C57/BL/6 background were the progeny of mice originally obtained from The Jackson Laboratory (Bar Harbor, MA, USA). All mice were housed under specific pathogen free (SPF) conditions at the ALAAC accredited animal facility at the Panum Institute (Copenhagen, Denmark) and were allowed to rest for at least one week before entering an experiment.

### 2.2. Virus Preparation and Quantitation

ZIKV, strain MR766 (Uganda, 1947), was obtained from American Type Culture Collection (ATCC) (Manassas, VA, USA) and propagated in Vero cells (ATCC CCL-81) grown in DMEM medium (supplemented with 10% FBS, NaHCO_3_, L-glutamine, Na-pyruvate and, penicillin and streptomycin). The titer of the virus stock was determined based on the number of plaque forming units (pfu) in semi-confluent monolayers of Vero cells. Specifically, 10-fold serial dilutions of the virus stock were prepared and incubated for 2 h on Vero cell monolayers that had been seeded one day earlier in 24-well plates. After the 2 h incubation, cells were overlaid with medium (containing 0.9% methylcellulose) and were further incubated for five days (37 °C, 5% CO_2_). After fixation with 4% formaldehyde, cells were stained with 0.1% crystal violet for plaque visualization.

For quantitation of virus in the organs of mice, the organs were first homogenized in PBS to yield 10% organ suspensions and viral titers were subsequently determined as described above. The detection limit of the assay was 250 pfu/g of organ.

### 2.3. Recombinant Adenoviral Vectors

E1-deleted E3 inactivated, human serotype 5 recombinant adenoviral (Ad5) vectors expressing the Zika virus non-structural proteins NS1/NS2 fused to the MHC class II-associated invariant chain (Ii) (Ad-liNS1/NS2), or not (Ad-NS1/NS2), were designed based on the ZIKV MR766 sequence and synthesized by VectorBuilder (Neu-Isenburg, Germany). Vector design followed the same principles as described previously [26,27]. The adenoviral vectors were purified using standard methods and the insert was verified by sequencing. The titers of the vaccine stocks were determined using Adeno-X Rapid Titer Kit (Clontech Laboratories, Mountain View, CA, USA).

### 2.4. Immunization and Viral Intracranial Challenge

The vaccine solutions were prepared by appropriately diluting the vaccine stocks in phosphate-buffered saline (PBS). For the Ad-ZIKV liNS1/NS2 and Ad-ZIKV NS1/NS2 constructs, mice received 2 × 10^7^ pfu/30 µL s.c. in the right footpad. For immunization with ZIKV MR766, mice received 1 × 10^3^ pfu/300 μL i.v.

For the intracranial (i.c.) challenge, mice received 1 × 10^3^ pfu or 5 × 10^2^ pfu/30 μL ZIKV MR766. Health status and weight were monitored daily or on every other day after i.c. challenge, and mice were euthanized when severe signs of illness along with a weight loss exceeding 25% of the initial weight were recorded.

Prior to vaccination and i.c. challenge, mice were sedated with isoflurane.

### 2.5. Anti-ZIKV NS1 Antibody Response

ZIKV-NS1 antibody levels in the serum of infected or naive mice were examined using a commercial kit (XpressBio, cat.no. IM-400C). Serum samples were diluted 50 times and were processed according to the manufacturer’s instructions on 96-well plates pre-coated with mouse ZIKV NS1 proteins, and finally, absorbance at 405 nm was measured on an ELISA plate reader (Thermo Scientific Multiskan FC, cat. no. N07710).

### 2.6. Flow Cytometry Analyses

Spleens were removed aseptically and transferred to Hanks Balanced Salt Solution (HBSS). Single-cell suspensions were obtained by pressing the spleens through a 70 µm nylon cell strainer, followed by centrifugation and two washes in HBSS, before re-suspension in RPMI 1640 cell culture medium containing 10% FCS supplemented with NaHCO_3_, 2-ME, L-glutamine, and penicillin-streptomycin. Approximately 2 × 10^6^ splenocytes were transferred to U-bottom 96-well microtiter plates and incubated for 5 h (37 °C, 5% CO_2_) with 70 μL RPMI 1640 cell culture medium (containing 1% L-glutamin, 1% penicillin, 1% streptomycin, 1% 2-mercaptoethanol (2-ME), and 10% fetal calf serum (FBS)), supplemented with 50 μL IL-2 (50 IU/mL), 50 μL Monensin (2 μg/mL), and 30 μL (1 μg/mL) of the relevant peptide for stimulation. Control samples did not receive any peptide. For demonstration of degranulation, anti-CD107α was included in the culture medium during the incubation. Following incubation, the cells were centrifuged (2000 rpm, 3 min), washed with FACS buffer with Monensin (PBS containing 1% BSA, 0.1% NaN3, and 3 μM Monensin) and incubated for 20 min (4 °C, dark) with 50 μL FACS/Monensin medium containing the relevant surface antibodies (1:100). Cells were then washed twice with PBS/Monensin medium (3 μM Monensin in PBS) and fixed in 100 μL 2% paraformaldehyde (PFA) and 100 μL PBS/Monensin for 15 min (4 °C, dark). Afterward, cells were washed with FACS/Monensin medium and incubated for 10 min (20 °C, dark) with 200 μL Saponin medium (PBS containing 0.5% Saponin). Next, the cells were incubated for 20 min (4 °C, dark) with 50 μL Saponin medium containing the relevant antibodies for intracellular staining (1:100). The cells were subsequently washed twice with Saponin medium and finally resuspended in FACS/Monensin medium and stored at 4 °C until flow cytometry analysis. Cell samples were analyzed using FACS LSRFortessa (BD Biosciences, Franklin Lakes, NJ, USA) and the data were analyzed using FlowJo software version 10 (BD Biosciences, Franklin Lakes, NJ, USA).

### 2.7. Peptide and Antibodies

For demonstration of virus-specific CD8 T cells, the ZIKV NS2B_1478–1486_: ICGMNPIAI peptide was used for stimulation. The following fluorochrome-conjugated monoclonal Abs were used for flow cytometry: for surface staining: α-CD44–FITC, α-CD8–PerCP-Cy5.5, α-CD44–APC/Cy7, and α-CD107α–ALEXA 488; for intracellular cytokine staining (ICS): α-IFNγ–APC, α-TNFα–PE/Cy7, and α-IL-2–PE. All antibodies were purchased from Biolegend as anti-mouse antibodies. To prevent unspecific binding, the anti-CD16/32 antibody was included in all staining setups.

### 2.8. In Vivo CD8 T Cell Depletion

The InVivoMab anti-mouse CD8a (YTS 169.4) purchased from BioXcell was used for in vivo depletion of CD8 T cells. Mice to be depleted were injected i.p. with 200 μg of the antibody one day prior to i.c. challenge and with 100 μg of antibody one and four days post challenge. The efficiency of the cell depletion was confirmed by flow cytometric analysis of the splenocytes.

### 2.9. Serum Transfer

Serum was collected from mice immunized with 2 × 10^7^ pfu/30 µL of Ad-liNS1/NS2 s.c. four weeks earlier and transferred to naive recipient mice. Mice received 500 µL serum i.v. and 500 µL serum i.p. three days prior to i.c. challenge and 300 µL serum i.v., again one day before i.c. challenge.

### 2.10. Statistical Evaluation

GraphPad Prism Software (version 7) was used for the statistical analysis. For comparison of multiple datasets, a Kruskal–Wallis test (one-way ANOVA test, non-parametric) was initially performed and if groups differed significantly, pairwise comparisons were subsequently carried out using a non-parametric Mann–Whitney U-test. A *p*-value of <0.05 was considered evidence of a statistically significant difference.

## 3. Results

### 3.1. Adenovirus Vectors Expressing ZIKV NS1/NS2 with and without Li-Linkage

**Figure 1:**
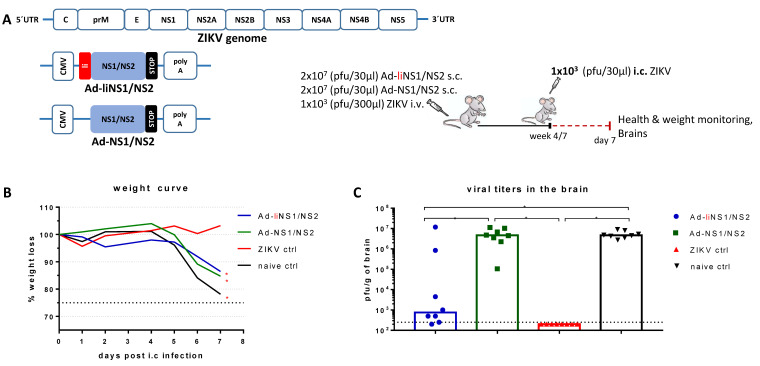
Level of protection conferred by Ad-liNS1/NS2 and Ad-NS1/NS2 vaccines. (**A**) Graphical representation of the ZIKV genome organization and the ZIKV proteins (NS1/NS2) encoded by the recombinant adenoviral vectors, with and without li-linkage, under the control of the Cytomegalovirus (CMV) promoter. (**B**) WT C57BL/6 mice were vaccinated s.c. with 2 × 10^7^ pfu of either vaccine (Ad-liNS1/NS2 and Ad-NS1/NS2) and four or seven weeks later, these mice, along with ZIKV-immune and naive mice, were challenged i.c. with 1 × 10^3^ pfu ZIKV. Mice were weighed and monitored daily. (**C**) On day 7 post i.c. challenge, brains were removed and viral titers were determined by a standard plaque assay. The detection limit for virus in brain was 250 pfu/g organ and is displayed as a stippled line. Each dot represents an individual animal. The columns represent the group medians and data are pooled from two independent experiments. * *p* < 0.05. For the weight curves, error bars were omitted for clarity, instead, stars denote a statistical significant difference compared to the unvaccinated control group.

We designed two replication-deficient adenoviral vectors targeting the ZIKV non-structural proteins 1 and 2 (NS1/NS2); one encoding a NS1/NS2 fusion protein (Ad-NS1/NS2) and one encoding the same NS1/NS2 antigen covalently coupled to the MHC-II associated invariant chain (Ad-liNS1/NS2). The vectors were designed based on the ZIKV MR766 sequence. To secure optimal antigen expression, we included the constitutive cytomegalovirus (CMV) promoter at the beginning of the transgenic region and a polyadenylation signal at the end, as shown in Figure 1A.

### 3.2. Quality of Protection Conferred by the Ad-liNS1/NS2 and Ad-NS1/NS2 Vaccines

To assess the protective efficacy of our two vaccines, we utilized a mouse model previously developed by our group [6]. Based on our model, we showed that peripheral ZIKV administration led to asymptomatic ZIKV infection, which can ultimately confer immunity against subsequent lethal intracranial (i.c.) challenge with ZIKV.

To test our vaccines, wild type (WT) immunocompetent mice received 2 × 10^7^ pfu of either the Ad-liNS1/NS2 or the Ad-NS1/NS2 vaccine subcutaneously (s.c.). Naive mice and mice receiving ZIKV intravenously (i.v.) were also included as controls. After four or seven weeks, all mice were challenged i.c. with 1 × 10^3^ pfu of ZIKV and their health and weights were monitored for a week. On day 7 post challenge, brains were removed and the viral load was measured via a plaque assay.

We observed that while both vaccinated groups experienced acute weight loss after day 5 post i.c. (similar to the naive control group), the mice in the group receiving the Ad-liNS1/NS2 construct maintained a healthier phenotype (mice were more active than in the other groups and there was less ruffling of the fur) and had significantly lower levels of detectable replicating virus in the brain (Figure 1B,C) than the naïve controls. As expected [6], mice that were immunized with ZIKV i.v. were protected and we did not find viral replication in their brains.

### 3.3. Immunogenicity of Ad-liNS1/NS2 and Ad-NS1/NS2 Vaccines

**Figure 2:**
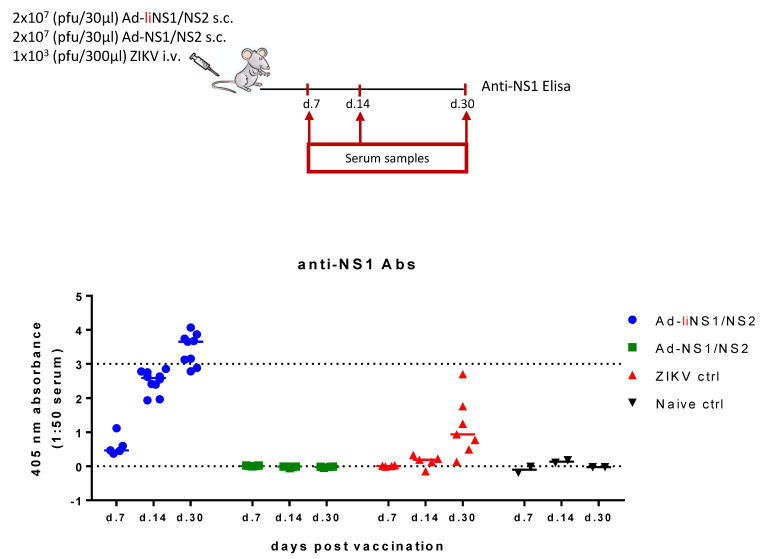
Anti-NS1 antibody levels induced by the Ad-liNS1/NS2 and Ad-NS1/NS2 vaccines. WT C57BL/6 mice were vaccinated s.c. with 2 × 10^7^ pfu of either vaccine (Ad-liNS1/NS2 and Ad-NS1/NS2). ZIKV-immune and naive mice were included for control. On days 7, 14, and 30 post vaccination, serum samples were collected and the levels of NS1-antibodies were measured based on an ELISA kit. Each dot represents an individual animal. The vertical bars represent the group medians and the stippled lines the assay sensitivity.

**Figure 3:**
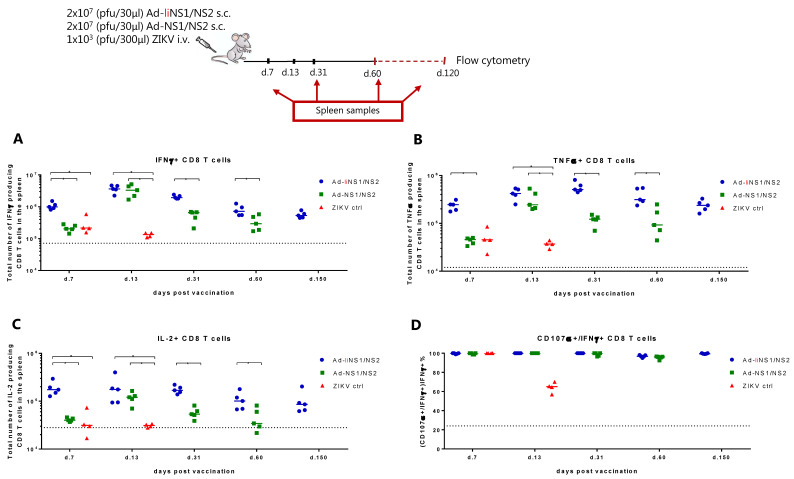
CD8 T cell response induced by the Ad-liNS1/NS2 and Ad-NS1/NS2 vaccines. WT C57BL/6 mice were vaccinated s.c. with 2 × 10^7^ pfu of either vaccine (Ad-liNS1/NS2 and Ad-NS1/NS2). ZIKV-immune and naive mice were included for control. On days 7, 13, 31, 60, and 150 post vaccination, spleens were harvested and CD8 T cells were examined by ICS (for details, see M & M) for the total numbers of IFNγ-producing CD8 T cells (**A**), total numbers of TNFα-producing CD8 T cells (**B**), and total numbers of IL-2-producing CD8 T cells (**C**) as well as the percentage of IFNγ-producing CD8 T cells co-producing CD107α (**D**) following ex vivo peptide stimulation with NS2B_1478-1486_. Each dot represents an individual animal. The bars represent the group medians and the stippled line background. * *p* < 0.05. For representative flow plots, see Supplementary Figures S1 and S2.

Naturally, we wondered what caused the difference in the virus control levels conferred by our two vaccines. We hypothesized that the answer lay in the type of the immune response elicited by each construct. To evaluate the primary immune response to each of our vaccines, WT mice received 2 × 10^7^ pfu of either the Ad-liNS1/NS2 or the Ad-NS1/NS2 vaccine s.c. and at specified timepoints, the anti-NS1 antibody levels in the serum (Figure 2) and the CD8 T cell response in the spleen (Figure 3) were investigated.

To check the antibody response, we used a commercially available ELISA kit to measure the anti-NS1 antibody levels on days 7, 14, and 30 following vaccination with each vaccine. A group of mice receiving ZIKV i.v. and naive mice were included as controls. We observed that anti-NS1 antibodies were robustly induced and increased over time in the serum of mice vaccinated with the Ad-liNS1/NS2 construct (Figure 2). In contrast, at all timepoints tested, the levels of anti-NS1 antibodies in the serum of Ad-NS1/NS2 mice were undetectable and similar to the response in the naive serum. Infection with ZIKV i.v. induced a weak anti-NS1 antibody response detectable on day 30.

To assess the CD8 T cells response, spleens were harvested on days 7, 13, 31, and 60 following vaccination with each adeno-construct, and the ability of CD8 T cells to produce cytokines IFNγ, IL-2, and TNFα following ex vivo peptide stimulation with NS2B_1478-1486_ was measured by flow cytometry. For the Ad-liNS1/NS2 construct, we additionally tested the CD8 T cell response on day 150 post vaccination. We observed that, on day 7 post vaccination, total numbers of IFNγ-producing CD8 T cells were significantly higher in the mice receiving the Ad-liNS1/NS2 vaccine than in mice receiving AdNS1/NS2 or infected with ZIKV (Figure 3A). By day 13, the Ad-NS1/NS2 vaccinated group reached similar numbers of IFNγ expressing cells as the Ad-liNS1/NS2 vaccinated group, but after day 31, the Ad-liNS1/NS2 group again took the lead (Figure 3A). The expression of the rest of the tested cytokines (IL-2 and TNFα) followed a similar pattern to that observed regarding IFNγ expression (Figure 3B,C). In addition, we assessed the degranulation capacity of the induced antigen-specific CD8 T cells via measuring CD107α expression following peptide stimulation. We observed that CD8 T cells from both vaccinated groups could degranulate, thus all IFNγ-producing CD8 T cells were found capable of additionally expressing CD107α (Figure 3D). Representative plots can be found in Supplementary Figures S1 and S2.

It appeared that the Ad-liNS1/NS2 construct could induce a robust anti-NS1 antibody response in addition to a strong CD8 T cell response that was mounted faster and maintained at a higher level for a longer time. From this point onward, we focused our studies on the Ad-liNS1/NS2 vaccine.

### 3.4. Kinetic of Viral Control in Ad-liNS1/NS2 Vaccinated Mice

**Figure 4:**
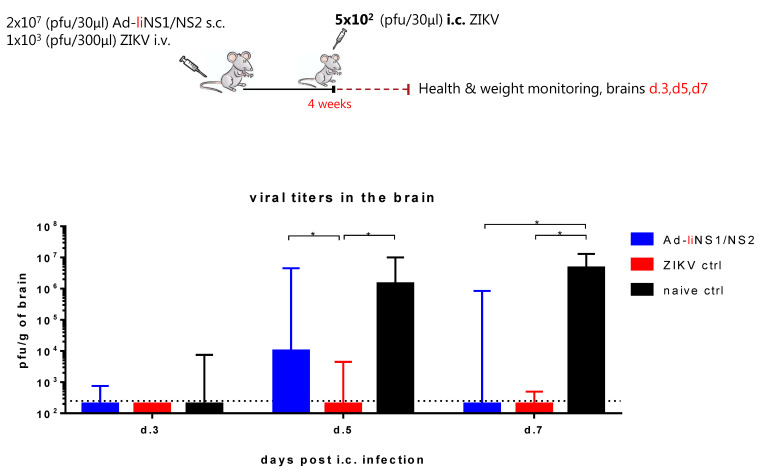
Kinetics of viral clearance in Ad-liNS1/NS2 vaccinated mice. WT C57BL/6 mice were vaccinated s.c. with 2 × 10^7^ pfu of either Ad-liNS1/NS2 and four weeks later, these mice, along with ZIKV-immune and naive mice, were challenged i.c. with 5 × 10^2^ pfu ZIKV. On days 3, 5, and 7 post i.c. challenge, brains were removed and viral titers were determined by a standard plaque assay. The detection limit for virus in brain was 250 pfu/g organ and is displayed as a stippled line. The results represent the group medians with range and are pooled from two independent experiments. * *p* < 0.05.

First, we wanted to assess the kinetics of virus control in the brains of vaccinated mice compared to immune and naive mice. As shown in Figure 1, vaccination with the Ad-liNS1/NS2 vaccine resulted in significantly lower viral loads in the brain of vaccinated mice compared to naive mice on day 7 post i.c. Nevertheless, the weight loss observed beyond day 5 after viral challenge suggested that the viral loads in the vaccinated mice peaked at a higher peak level compared to mice immunized through prior infection (the ZIKV immune control group) whose weights were unaffected following i.c. challenge. Therefore, we set out to investigate the kinetics of virus control in the brain of vaccinated mice. To that end, WT mice received 2 × 10^7^ pfu of the Ad-liNS1/NS2 vaccine s.c. and four weeks later, mice were challenged i.c. with 5 × 10^2^ pfu of ZIKV. Naive and ZIKV-immune groups were included for control. On days 3, 5, and 7 post i.c, brains were removed and the viral load was measured via a plaque assay. We observed that while on day 3 post i.c., the virus control was equally efficient in both vaccinated mice and in the mice immunized by previous ZIKV infection, by day 5 post i.c., there was significantly higher viral load in the vaccinated group (Figure 4). Still, the viral load in vaccinated mice on day 5 post i.c. was significantly lower than in the naive control group. By day 7 post i.c, the vaccinated group seemed to control the viral load almost as efficiently as the ZIKV-immune group.

Viral control in the ZIKV-immune group relies primarily on the induced nAbs which block viral replication early on [6]. The above results reveal that the immune response elicited by the Ad-liNS1/NS2 vaccine (anti-NS1 antibodies and CD8 T cell response) takes more time to affect the course of the infection, but nevertheless, eventually manages to control ZIKV replication in the brain.

### 3.5. Contribution of CD8 T Cells and Anti-NS1 Antibodies in Ad-liNS1/NS2 Mediated Protection

Next, we wanted to elaborate on the arm of the adaptive immunity contributing to the observed protection in the Ad-liNS1/NS2 vaccinated mice.

**Figure 5:**
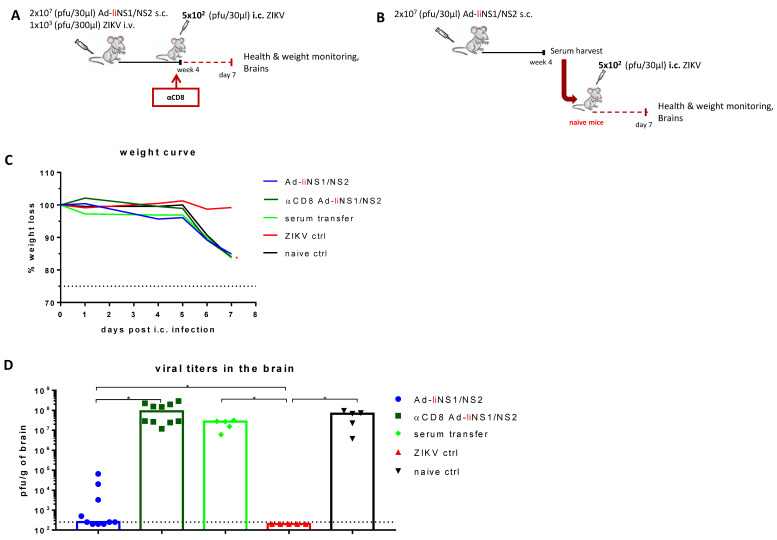
Contribution of CD8 T cells and anti-NS1 antibodies in Ad-liNS1/NS2 mediated protection. (**A**) Graphical representation of the experimental setup used to study the effect of CD8 T cells in protection. WT C57BL/6 mice were vaccinated s.c. with 2 × 10^7^ pfu of Ad-liNS1/NS2 and four weeks later, mice were depleted of CD8 T cells (αCD8-Ad-liNS1/NS2) prior to i.c. challenge with 5 × 10^2^ pfu ZIKV. A group of vaccinated, undepleted mice along with ZIKV-immune and naive mice were included for comparison. (**B**) Graphical representation of the experimental setup used to study the effect of anti-NS1 antibodies in protection. WT C57BL/6 mice were vaccinated s.c. with 2 × 10^7^ pfu of Ad-liNS1/NS2 and four weeks later, serum was harvested and transfused to naive recipient mice three and one day prior to i.c. challenge with 1 × 10^3^ pfu ZIKV. (**C**) All groups were weighed and monitored daily following i.c. challenge. (**D**) On day 7 post i.c. challenge, brains were removed, and viral titers were determined by a standard plaque assay. The detection limit for virus in brain was 250 pfu/g organ and is displayed as a stippled line. Each dot represents an individual animal. The columns represent the group medians and results for the αCD8 depletion set-up were pooled from two independent experiments. * *p* < 0.05. For the weight curves, error bars were omitted for clarity, instead, stars denote a statistical significant difference compared to the unvaccinated control group.

To evaluate the role of the induced anti-NS1 antibodies in protection, mice were vaccinated s.c. with 2 × 10^7^ pfu of the Ad-liNS1/NS2 vaccine and whole serum was harvested after four weeks. The serum was transfused into naive recipient mice, which were subsequently challenged with 5 × 10^2^ pfu of ZIKV i.c. Unlike what we have previously observed with serum from ZIKV immunized mice [6], we did not observe any benefit from the transfusion of serum from AdIiNS1/NS2 vaccinated animals, and the mice receiving anti-NS1 specific serum were unable to control viral replication, similar to the naive control group (Figure 5).

**Figure 6:**
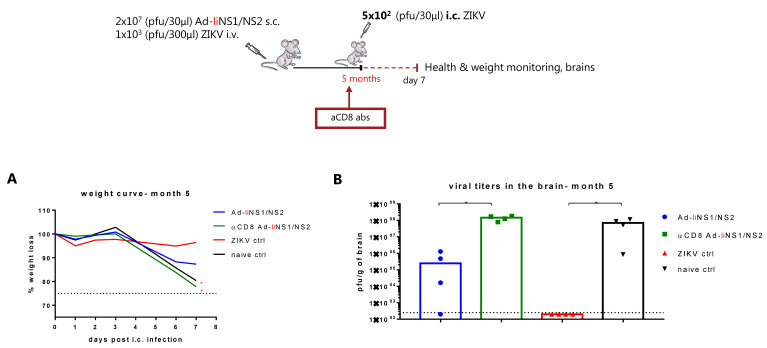
Role of CD8 T cells in long-term protection from the Ad-ZIKV liNS1/NS2 vaccine. WT C57BL/6 mice were vaccinated s.c. with 2 × 10^7^ pfu of Ad-liNS1/NS2 and five months later, mice were depleted of CD8 T cells (αCD8-Ad-liNS1/NS2) prior to i.c. challenge with 5 × 10^2^ pfu ZIKV. A group of vaccinated, undepleted mice along with ZIKV-immune and naive mice were included for comparison. (**A**) All groups were weighed and monitored daily following i.c. challenge. (**B**) On day 7 post i.c. challenge, brains were removed and viral titers were determined by a standard plaque assay. The detection limit for virus in brain was 250 pfu/g organ and is displayed as a stippled line. Each dot represents an individual animal. The columns represent the group medians. * *p* < 0.05. For the weight curves, error bars have been omitted for clarity, instead, stars denote a statistical significant difference compared to the unvaccinated control group.

To investigate the role of CD8 T cells, we vaccinated mice s.c. with 2 × 10^7^ pfu of the Ad-liNS1/NS2 vaccine and around the time of i.c. challenge, CD8 T cells were depleted via administration of the anti-CD8 antibody. We observed that removing this cell subset resulted in increased levels of ZIKV replication in the CNS, similar to that in the naive controls (Figure 5). Similar results were observed when CD8 T cells were depleted from mice vaccinated five months prior to i.c. challenge (Figure 6).

These results clearly indicate that CD8 T cells are essential for protection while anti-NS1 antibodies have minimal, if any, contribution on their own.

### 3.6. Potential Mechanism of Protection Mediated by the Ad-liNS1/NS2 and Ad-NS1/NS2 Vaccines

Considering that the difference in levels of NS1-specific antibodies was perhaps the most striking difference between mice vaccinated with the two vaccines, we were somewhat perplexed by the fact that serum from the Ad-liNS1/NS2 vaccinated mice, even though it contained robust anti-NS1 antibody levels, failed to confer any measurable degree of virus control to naive recipient mice (Figure 5). To explain our findings, we hypothesized that perhaps virus control required collaboration between antibodies and virus-specific CD8 T cells [22,31], and that the latter was lacking in the transfused naïve mice. To test this possibility, we selected to vaccinate WT mice with the Ad-NS1/NS2 vaccine to provide a base level of T-cell immunity. Part of these mice were then transfused with serum from Ad-liNS1/NS2 vaccinated mice rich in anti-NS1 antibodies prior to i.c. challenge. According to our hypothesis, the CD8 T cell response induced by the Ad-NS1/NS2 vaccine, which on its own was insufficient to confer protection (Figure 1) against i.c. challenge, would then be helped by the administration of anti-NS1 antibodies. In that scenario, we also wanted to evaluate the role of IFNγ, as previous analysis have pointed to the collaboration between antibodies and this cytokine in protection against ZIKV infection [32].

**Figure 7:**
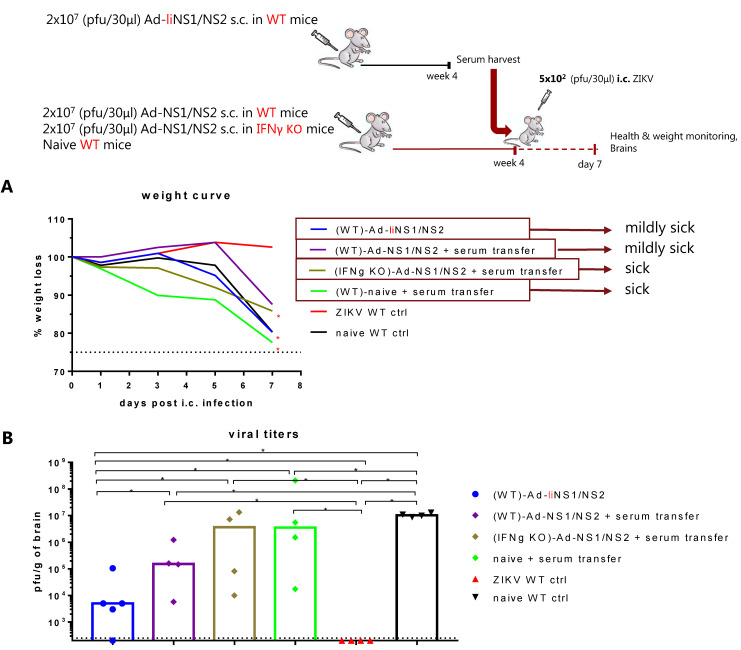
Mechanism of protection resulting from Ad-liNS1/NS2 vaccination. WT C57BL/6 mice were vaccinated s.c. with 2 × 10^7^ pfu of Ad-liNS1/NS2 and four weeks later, serum was harvested and transfused to Ad-NS1/NS2 vaccinated WT mice, Ad-NS1/NS2 vaccinated IFNγ KO mice, or naive recipient mice three and one day prior to i.c. challenge with 5 × 10^2^ pfu ZIKV. A group of ZIKV-immune and naive mice were included for control. (**A**) All groups were weighed and monitored daily following i.c. challenge. (**B**) On day 7 post i.c. challenge, brains were removed and viral titers were determined by a standard plaque assay. The detection limit for virus in the brain was 250 pfu/g organ and is displayed as a stippled line. Each dot represents an individual animal. The columns represent the group medians. * *p* < 0.05. For the weight curves, error bars were omitted for clarity, instead, stars denote a statistical significant difference compared to the unvaccinated control group.

Consequently, WT mice were vaccinated s.c. with 2 × 10^7^ pfu of the Ad-liNS1/NS2 vaccine and whole serum was harvested after four weeks. The serum was subsequently adoptively transferred to WT and IFNγ KO mice that were previously vaccinated with the Ad-NS1/NS2 vaccine. Naive mice receiving immune serum and Ad-liNS1/NS2 vaccinated mice along with groups of ZIKV immune and naive mice were included as controls. All mice were challenged i.c. with 5 × 10^2^ pfu of ZIKV and their health and weights were monitored for a week. On day 7 post i.c., brains were removed and the viral load was determined using a plaque assay. We observed that while serum transfer into unvaccinated mice again failed to significantly reduce the viral load in the CNS, a significant reduction was observed in WT mice vaccinated with Ad-NS1/NS2 and given the same serum. In this context, remember that prior vaccination with Ad-NS1/NS2 on its own did not have any effect on the viral load day 7 after challenge (see Figure 1C). Notably, mice in the corresponding IFNγ KO group had similar levels of virus in the CNS as the naive controls (Figure 7). These results are consistent with the hypothesis that there is a collaboration of CD8 T cells and antibodies in virus control and that IFNγ could be involved.

Nevertheless, there was still a significant difference in capacity for viral control between the Ad-liNS1/NS2 vaccinated group and the WT-Ad-NS1/NS2 vaccinated group receiving immune serum (Figure 7). This could suggest that despite the limited difference in the magnitude of the measured CD8 T cell response, perhaps the memory CD8 T cells induced by the Ad-liNS1/NS2 vaccine were better equipped to handle the viral challenge. Alternatively, a greater difference in the magnitude of the vaccine induced CD8 T cell response could exist with regard to T-cell specificities not investigated here.

## 4. Discussion

Given that the population receiving the ZIKV vaccine will include pregnant women, it is critical that the vaccine is non-replicating, but still immunogenic. In this study, we investigated the vaccination potential of two replication-deficient adenoviral vectors targeting the ZIKV non-structural proteins 1 and 2 (NS1/NS2); one carrying the free NS1/NS2 antigens (Ad-NS1/NS2) and one with the NS1/NS2 antigens covalently coupled to the invariant chain (Ad-liNS1/NS2). The invariant chain (li) is a small protein primarily involved in the presentation of exogenous peptides to CD4 T cells [25] and is a patented adjuvant developed by our group [26]. Previous results from our group have demonstrated that li-coupling in most cases augments and prolongs the virus-specific T-cell response, particularly against MHC class I-restricted epitopes [26].

The goal of vaccines is to induce protective immune responses without the pathogenic effects associated with natural infection with the live virus. We have previously established an immunocompetent mouse model to study protective immunity in the context of natural ZIKV infection [6]. Based on our model, we have demonstrated that natural ZIKV immunity can confer high levels of protection against lethal i.c. challenge. To assess whether the levels of protection conferred by our adeno-vaccines matched the levels conferred by natural ZIKV infection, we immunized WT C57BL/6 mice s.c. in the footpad with each vaccine and 4–7 weeks later, these mice, along with the ZIKV-immune and naive control mice, were challenged i.c. with ZIKV. Mice were monitored for seven days following i.c. infection and health and weight were registered daily (Figure 1B). While monitoring the weight following challenge revealed that none of the vaccines were able to prevent weight loss, the group receiving the Ad-liNS1/NS2 construct did not experience severe symptoms of the disease and maintained a relatively healthy phenotype throughout the week (personal observation, not shown). When we checked the viral loads in the brains on day 7 post i.c, it was evident that mice vaccinated with Ad-liNS1/NS2 had a better viral control in the CNS than mice vaccinated with Ad-NS1/NS2 (Figure 1C).

Next, we wanted to assess the types of immune responses induced by the vaccines and hoped to identify the features that rendered the Ad-liNS1/NS2 construct superior. We compared the phenotype of the CD8 T cells induced by each of the Ad-liNS1/NS2 and Ad-NS1/NS2 vaccines in the effector and memory phase and found that, overall, delivering the antigens of interest with the adenovector system increased the CD8 T cell response to that antigen compared to infection with live ZIKV. However, it was clear that linking the antigen to the li induced an accelerated response that was sustained at high levels over time (Figure 3). Nevertheless, it should be noted that we measured the CD8 T cell response against a single epitope in the NS2B region, so we do not know whether other epitopes exist and the magnitude of the response they may induce. The perhaps most notable effect of li-linkage was on the induction of anti-NS1 antibodies. The serum of Ad-liNS1/NS2 vaccinated mice contained a striking amount of antibodies compared to the Ad-NS1/NS2 group where we could only detect background levels (Figure 2).

Ultimately, we placed our focus on deciphering the protection mediated by the Ad-liNS1/NS2 vaccine. We started by comparing the kinetics of virus control in the CNS of Ad-liNS1/NS2 vaccinated mice to that of ZIKV-immune and naive mice. We found that while the ZIKV-immune mice were better at controlling viral replication from the start, it took more time for the Ad-liNS1/NS2 vaccinated mice to gain control. The reason for this difference is likely to be found in the profile of the immune response elicited in each group; peripheral infection with ZIKV induces potent nAbs (which has been proven efficient in viral control), while the Ad-liNS1/NS2 vaccine induces non-neutralizing anti-NS1 antibodies and a polyfunctional CD8 T cell response.

Next, we assessed the role of the induced anti-NS1 antibodies in protection by performing adoptive transfer of immune serum to naive recipient mice and thereafter checked their ability to control an i.c. ZIKV infection. To our surprise, but consistent with recently published data [22], we did not detect any improvement in viral control in naive recipient mice (Figure 5). However, we noticed that when there were suboptimal levels of cytotoxic CD8 T cells (as induced by the Ad-NS1/NS2 vaccine), anti-NS1 antibody transfer could perhaps assist in viral control (Figure 7). Still, the degree of protection did not match that of the Ad-liNS1/NS2 vaccinated mice, which implies that optimal priming of a CD8 T cell response is critical. The important role of CD8 T cells in protection was clearly established when we performed in vivo CD8 T cell depletion in Ad-liNS1/NS2-vaccinated mice and observed that these mice were left unprotected against i.c. challenge (Figure 5 and Figure 6). These data collectively highlight that CD8 T cells are indispensable for the protection conferred by the Ad-liNS1/NS2 vaccine, in contrast to the anti-NS1 abs, which alone failed to impact viral loads in the recipient mice.

Overall, our study demonstrates that vaccination with Ad-liNS1/NS2 is able to confer increased levels of protection against subsequent ZIKV challenge in immunocompetent adult mice. Our data suggest that li-linkage of the ZIKV NS1/NS2 antigens significantly accelerates the production of polyfunctional CD8 T cells, but also augments the production of anti-NS1 antibodies, and underscores the potential of this strategy to result in an effective and safe vaccine against ZIKV without the risk of ADE. However, development of NS1 vaccines may not be without significant difficulties, as recent studies in mice infected with high doses of Zika virus or vaccinated with NS1 protein have revealed the appearance in serum of IgG antibodies reactive against multiple self-antigens in brain and muscles [33]. The importance of this observation is not yet clear, as there is no evidence that these antibodies are pathogenic nor that the same will happen in humans. Nevertheless, while the flavivirus NS1 protein represents a promising antigen, the issue of self-reactive antibodies needs to be carefully addressed.

## Data Availability

Not applicable.

## Supplementary Materials

The following are available online at https://www.mdpi.com/article/10.3390/v13112215/s1, Figure S1: Representative plots: Ad-liNS1/NS2 kinetics, Figure S2: Representative plots: Ad-NS1/NS2 kinetics.
