# Supplementary material for: Efficient Control of Zika Virus Infection Induced by a Non-Replicating Adenovector Encoding Zika Virus NS1/NS2 Antigens Fused to the MHC Class II-Associated Invariant Chain
Source: Viruses. 2021 Nov 3;13(11):2215. doi: 10.3390/v13112215 (PMC8625593; doi:10.3390/v13112215)

Figure S1: Representative plots: Ad-liNS1/NS2 kinetics

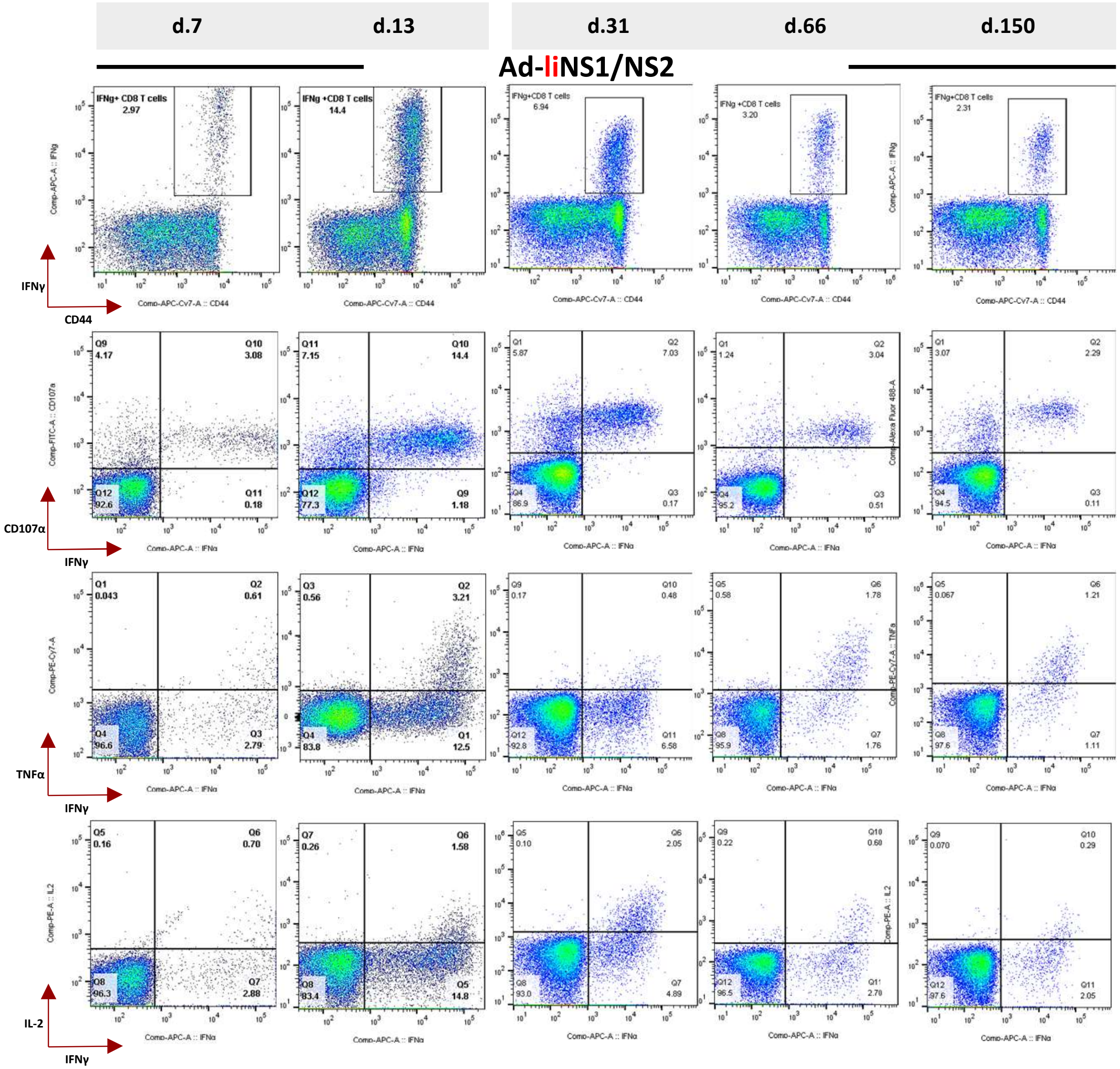

Figure S2: Representative plots: Ad-NS1/NS2 kinetics

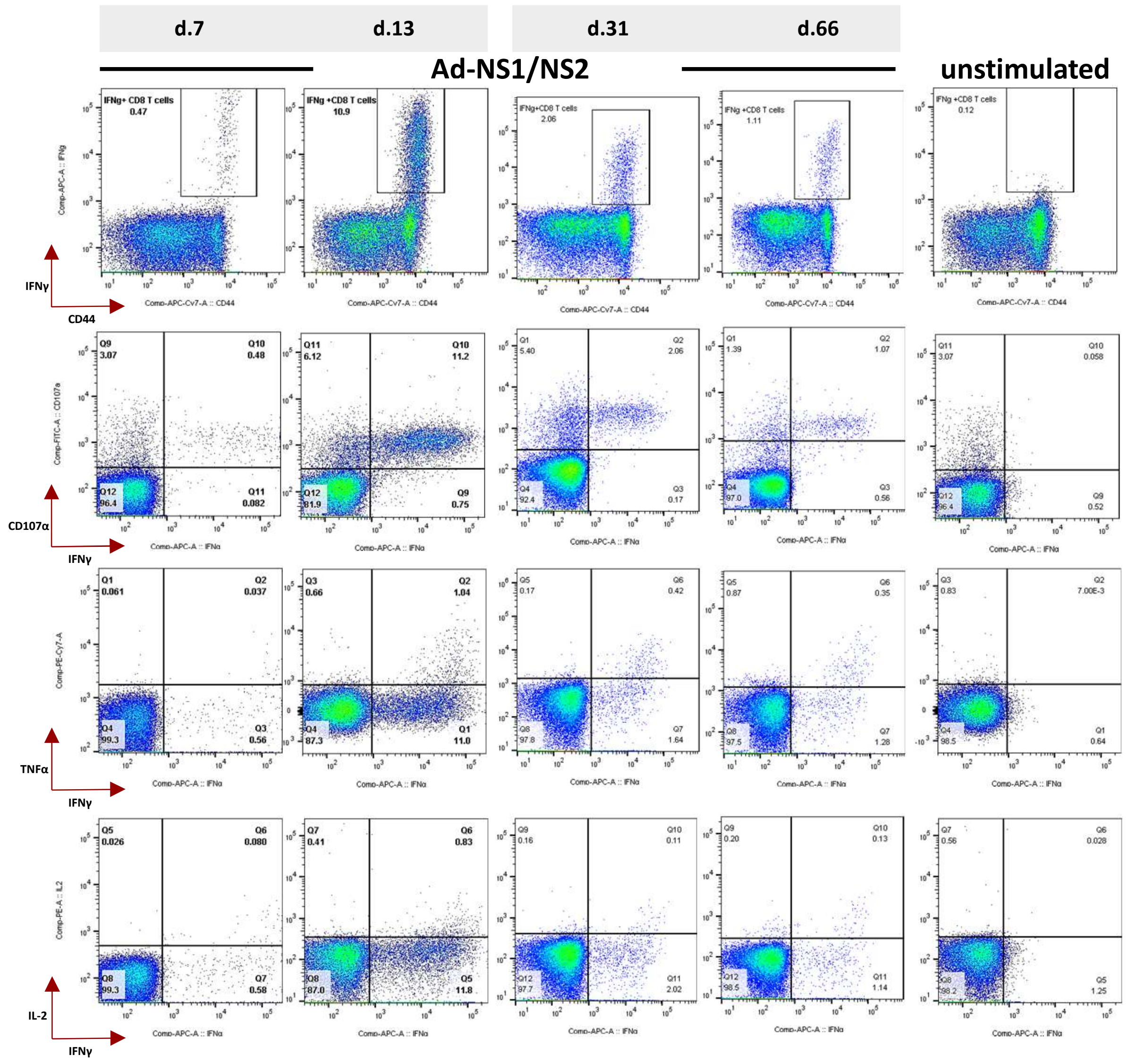

Supplement: Supplementary file 1 [file viruses-13-02215-s001.zip › viruses-1411170-supplementary.pdf]
